# Identification of Two *DNMT3A* Mutations Compromising Protein Stability and Methylation Capacity in Acute Myeloid Leukemia

**DOI:** 10.1155/2019/5985923

**Published:** 2019-10-30

**Authors:** Samantha Bruno, Maria Teresa Bochicchio, Eugenia Franchini, Antonella Padella, Giovanni Marconi, Andrea Ghelli Luserna di Rorà, Claudia Venturi, Maddalena Raffini, Giovanna Prisinzano, Anna Ferrari, Lorenza Bandini, Valentina Robustelli, Martina Pazzaglia, Maria Chiara Fontana, Chiara Sartor, Maria Chiara Abbenante, Cristina Papayannidis, Simona Soverini, Emanuela Ottaviani, Giorgia Simonetti, Giovanni Martinelli

**Affiliations:** ^1^Department of Experimental, Diagnostic and Specialty Medicine, University of Bologna, Institute of Hematology “L. e A. Seràgnoli”, Bologna, Italy; ^2^Istituto Scientifico Romagnolo per lo Studio e la Cura dei Tumori (IRST) IRCCS, Meldola (FC), Italy; ^3^Casa Sollievo della Sofferenza, San Giovanni Rotondo, Italy

## Abstract

Somatic mutations of *DNMT3A* occur in about 20% of acute myeloid leukemia (AML) patients. They mostly consist in heterozygous missense mutations targeting a hotspot site at R882 codon, which exhibit a dominant negative effect and are associated with high myeloblast count, advanced age, and poor prognosis. Other types of mutations such as truncations, insertions, or single-nucleotide deletion also affect the *DNMT3A* gene, though with lower frequency. The present study aimed to characterize two *DNMT3A* gene mutations identified by next-generation sequencing (NGS), through analysis of protein stability and DNA methylation status at CpG islands. The first mutation was a single-nucleotide variant of *DNMT3A* at exon 20 causing a premature STOP codon (c.2385G > A; p.Trp795^*∗*^; NM_022552.4). The *DNMT3A* mutation load increased from 4.5% to 38.2% during guadecitabine treatment, with a dominant negative effect on CpG methylation and on protein expression. The second mutation was a novel insertion of 35 nucleotides in exon 22 of *DNMT3A* (NM_022552.4) that introduced a STOP codon too, after the amino acid Glu863 caused by a frameshift insertion (c.2586_2587insTCATGAATGAGAAAGAGGACATCTTATGGTGCACT; p. Thr862_Glu863fsins). The mutation, which was associated with reduced DNMT3A expression and CpG methylation, persisted at relapse with minor changes in the methylation profile and at protein level. Our data highlight the need to better understand the consequences of *DNMT3A* mutations other than R882 substitutions in the leukemogenic process in order to tailor patient treatments, thus avoiding therapeutic resistance and disease relapse.

## 1. Introduction

Genetic alterations of chromatin regulators and epigenetic modifications cooperate to the pathogenesis of acute myeloid leukemia (AML) [1]. Among epigenetic modifications, DNA methylation represents a mechanism adopted by cells to regulate DNA accessibility through the conversion of 5-methylcytosine (5-mC) to 5-hydroxymethylcytosine (5-hmC). It switches on/off the promoter of several target genes, thus regulating their expression levels and contributing to disease phenotype. DNA methyltransferases (DNMT3A, DNMT3B, DNMT1, and DNMT3L) are the key enzymes involved in DNA methylation. DNMT3A and DNMT3B produce *de novo* hemimethylated DNA and *de novo* symmetric DNA methylation, respectively, while DNMT1 is involved in maintenance of DNA methylated status [2, 3].

DNA methylation has been identified as a specific functional category of mutated genes in AML that includes mutation in *DNMT3A*, *DNMT3B*, *DNMT1*, *Tet methylcytosine dioxygenase 1* and *2* (*TET1*, *TET2*), and *isocitrate dehydrogenase 1* and *2* (*IDH1*, *IDH2*) genes [4]. Moreover, *Wilms tumor 1* (*WT1*) mutations were shown to reduce DNA methylation levels significantly in AML patients, conferring the same hypomethylation signature observed in patients harbouring *TET2* mutations [5]. Haploinsufficiency of *WT1* in preclinical models was also shown to decrease 5-hmC levels and impair TET2 function, especially in elderly animals [6].

*DNMT3A* mutations are among the most frequent driver mutations in AML, third for frequencies to *Fms-like tyrosine kinase 3* (*FLT3*) and *nucleophosmin 1* (*NPM1*) [1]. About 20% of patients with *de novo* AML show recurrent somatic mutations in the *DNMT3A* gene [7], which represents an early lesion in leukemia development. Indeed, *DNMT3A* genomic alterations are considered a preleukemic event in hematopoietic stem cells (HSCs) and confer a proliferation advantage [8]. They have been identified in preleukemic stem cells of myelodysplastic syndrome (MDS) and AML patients [9]. Moreover, mutations in genes encoding epigenetic modifiers, including *TET2*, *ASXL transcriptional regulator 1* (*ASXL1*), and *DNMT3A* have been reported in aging healthy people and are considered the first “hit” for the “clonal hematopoiesis of indeterminate potential (CHIP)” [10, 11].

Genomic lesions of the *DNMT3A* gene mostly consist in missense mutations targeting a hotspot site at R882 codon, which exhibits a dominant negative effect [12]. Furthermore, *DNMT3A* R882 mutations are associated with a hypomethylated status of CpG islands, higher relapse rate, and poor prognosis [13–18], and they persist during remission [19, 20]. It has been recently shown that the methylation levels are dependent on the amount of active DNMT3A and higher methylation confers a better prognosis to AML patients [21]. Among patients carrying *DNMT3A* mutations, 15–20% do not show the substitution at R882 codon, but they harbour truncations, insertion, or single-copy deletions [1, 7].

The present study aimed to characterize two undescribed *DNMT3A* mutations identified by next-generation sequencing (NGS) in two different AML patients, through analysis of protein stability and DNA methylation status at CpG islands.

## 2. Materials and Methods

### 2.1. Patients

Samples were obtained from AML patients after informed consent was approved by the Institutional Ethical Committee (protocol 112/2014/U/Tess of Policlinico Sant'Orsola-Malpighi) in accordance with the Declaration of Helsinki.

### 2.2. Isolation of Mononuclear Cells

Bone marrow (BM) mononuclear cells were isolated by Ficoll density-gradient (Amersham Biosciences) and lysed in guanidine-thiocyanate-containing lysis buffer (RLT, Qiagen, Ltd).

### 2.3. Genomic DNA, RNA, and Protein Extraction

The AllPrep DNA/RNA/Protein Mini Kit (Qiagen, Ltd) was used to extract DNA, RNA, and protein from primary mononuclear cells according to the manufacturer's instructions.

### 2.4. Ion Torrent Next-Generation Sequencing and Variant Calling

The patients' mutational profile was determined using Oncomine Myeloid Research Assay. The libraries were quantified using the Ion Library TaqMan TM Quantitation Kit (Thermo Fisher Scientific) following manufacturer's instructions and run in the Ion 530™ chip on the Ion Torrent S5 instrument (Thermo Fisher Scientific). Sequence alignment and analysis were performed using the Ion Torrent Suite Software v.5.8.0 and the Ion Reporter software v.5.10.3.0 and v.5.10.5.0 (Thermo Fisher Scientific). Human genome build 19 (Hg 19) was used as the reference for sequence alignment. A minimum coverage depth per amplicon of 500 was required; nonsynonymous mutations with a variant allele frequency (VAF) ≥2.5% were reported; 5′ and 3′ untranslated regions (UTRs), intronic donor splice-site variants, and polymorphisms were filtered out.

### 2.5. Amplification and Sanger Sequencing

*DNMT3A* mutations were confirmed by Sanger sequencing. Briefly, reverse transcription was performed starting from 1 *μ*g of RNA using reverse transcriptase M-MuLV enzyme (Sigma-Aldrich®). The obtained cDNA was used to perform polymerase chain reaction (PCR) using Applied Biosystem AmpliTaq Gold® (Thermo Fisher Scientific) and the following primers: FW: 5′-TCGAGTCCAACCCTGTGATG-3′ and REV: 5′-TAACTTTGTGTCGCTACCT CAGTT-3′. Cycling conditions were as follows: 10 minutes at 95°C; 40 cycles: 30 seconds at 94°C; 45 seconds at 54°C; 30 seconds at 72°C; and 10 minutes at 72°C. PCR amplicons were purified using Applied Biosystems ExoSAP-IT™ PCR Product Cleanup Reagent (Thermo Fisher Scientific) according to manufacturer's instructions. Samples were sequenced according to dideoxy procedure BigDye® Terminator v3.1 Cycle Sequencing Kit (Thermo Fisher Scientific) on an Applied Biosystems 3730xL genetic analyzer system (Thermo Fisher Scientific).

### 2.6. Capillary Electrophoresis

The allelic ratio of *FLT3*-ITD was measured by capillary electrophoresis. The reaction was performed starting from 20 *μ*g of DNA, by using AmpliTaq™ Gold DNA polymerase enzyme, Buffer II, magnesium chloride (Thermo Fisher Scientific) and the following primers: forward, 5′-GCAATTTADGTATGAAAGCCAGC-3′, and reverse, 5′-CTTTCAGCATTTTGACGGCA ACC-3′. Cycling conditions were 10 minutes at 95°C; 30 seconds at 95°C; 60 seconds at 60°C; 60 seconds at 72°C for 35 cycles; 7 minutes at 72°C. The amplification products were sequenced on Applied Biosystems 3130 Genetic Analyzer (Thermo Fisher Scientific) and were analyzed with GeneMapper™ Software 5 (Thermo Fisher Scientific). The ratio of the area of mutated and wildtype (wt)-*FLT3* defined the allelic ratio.

### 2.7. Western Blot Analysis

Protein extracts were separated by sodium dodecyl sulphate-polyacrylamide gel electrophoresis (SDS-PAGE, Bio-Rad) and transferred onto nitrocellulose membranes. The following antibodies were used: rabbit anti-DNMT3A (D23G1; Cell Signaling Technologies) and goat anti-*β*-actin (Santa Cruz Biotechnology) as control; horseradish peroxidase (HRP)-conjugated anti-rabbit immunoglobulin (Ig)G (GE Healthcare) and anti-goat IgG (Santa Cruz) as secondary antibodies. ECL Prime (GE Healthcare) reagent was used for detection using ChemiDoc XRS+ System (Bio-Rad). Signal quantification was performed using Image J software.

### 2.8. DNA Methylation Assay

Methylation was quantified on total DNA using the MethylFlash Methylated DNA 5-mC Quantification Kit (Epigentek) using triplicates of 100 ng of DNA from each sample. The absorbance (OD) was read at 450 nm using the Multiskan EX automatic microplate reader (Thermo Fisher Scientific). To quantify the absolute amount of methylated DNA, we generated a standard curve using a negative control (ME3) and 5 dilutions of positive control (10.0; 5.0; 2.0; 1.0; 0.5 ng/*μ*l); next we determined the slope (OD/ng) using linear regression. According to manufacturer's instructions, we used the following formulas to calculate the amount and percentage of 5-mC:

(1) $$\begin{array}{c} 5\text{ mC}\left(\text{ng}\right)=\frac{\left(\text{sample OD}-\text{ ME}3\text{ OD}\right)}{\text{slope}\times 2^{*}},\\ 5\text{ mC}\left(\text{\%}\right)=\frac{5\text{mC}\left(\text{ng}\right)}{\text{sample DNA}\left(\text{ng}\right)\times 100}, \end{array}$$

Where ^*∗*^2 is a factor to normalize the positive control because it contains only 50% of 5 mC.

## 3. Results and Discussion

### 3.1. Identification of a Premature Stop Codon in Exon 20 of the *DNMT3A* Gene in AML

**Figure 1:**
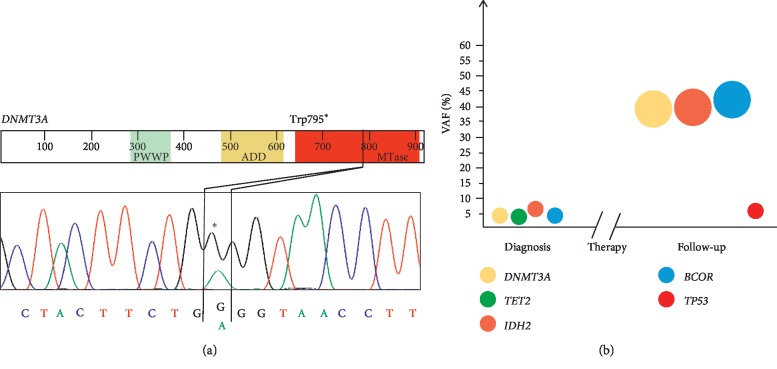
Sanger sequencing of the *DNMT3A* single-nucleotide variant identified in AML#1 and analysis of clonal evolution based on NGS data. (a) AML#1 showed a single-nucleotide variant of *DNMT3A* at exon 20 causing a premature STOP codon (c.2385G > A; p.Trp795^*∗*^; NM_022552.4). (b) Variant allele frequency (VAF) changes of the detected mutations at diagnosis and follow-up (after 4 courses of guadecitabine therapy), showing expansion of the *DNMT3A*-mutated clone.

NGS on a primary AML sample identified a single-nucleotide variant of *DNMT3A* at exon 20 causing a premature STOP codon (c.2385G > A; p.Trp795Ter) that was confirmed by Sanger sequencing (Figure 1(a)). Mutations at codon 795 were previously reported in angioimmunoblastic T-cell lymphoma (p.Trp795_Gly796ins3) [22] and in refractory anemia evolving to secondary AML (Trp795Cys) [23]. A STOP codon at position 795 has been reported in the Leiden Open Variation Database (c.2384G > A, https://www.lovd.nl/) and the c.2385G > A mutation has been found annotated in the database of single-nucleotide polymorphisms (dbSNP, rs1395575712). Moreover, missense variants affecting the codon 795 (chr2:25462024:A > C and chr2:25462024:A > G) were also annotated in the Genome Aggregation Database (gnomAD) with an allelic frequency of 0.000003979 and 0.000007957, respectively (https://gnomad.broadinstitute.org/). We detected the mutation (VAF = 4.5%) in the BM sample of a 74-year-old woman (AML#1), with 70% of AML blasts at diagnosis, normal karyotype, wt-*FLT3*, *NPM1*, and *tumor protein p53* (*TP53*) and intermediate cytogenetic risk according to ELN 2017 [24]. Mutations of *IDH2* (c.515G > A; p.Arg172Lys), *BCL6 corepressor* (*BCOR*) (c.2915_2916insA; p.Tyr972Ter), and *TET2* (c.3641G > A; p.Arg1214Gln) were also detected in the patient, with a VAF of 6.1%, 4.8%, and 3.2%, respectively (Figure 1(b) and Table 1). Previous evidence reported the co-occurrence of *DNMT3A* lesions with mutations of *NPM1*, *IDH2*, and *TP53* and with those affecting genes involved in chromatin and splicing in AML cases [25]. The patient received an induction therapy with the DNMT inhibitor guadecitabine [26], with persistence of disease at the bone marrow evaluation after 4 courses of therapy (40% of blasts and stable cytogenetic risk). The *DNMT3A* mutation load increased to 38.2% in the sample evaluated 4 months after treatment. NGS analysis revealed an increasing VAF during treatment of the *IDH2* (6.1% to 39.8%) and *BCOR* mutations (4.8% to 42.1%) detected at diagnosis, along with the emergence of a *TP53* mutation (c.607G > A; p.Val203Met, VAF 5.3%, Figure 1(b) and Table 1), which was not detectable at diagnosis. Nowadays, no predictive marker of response to guadecitabine has been defined for newly diagnosed AML. However, Chung et al. reported no significant association between gene mutations and complete remission in a cohort of 128 relapsed/refractory AML [27]. Trends were observed for *TET2*-mutated cases (higher CR rate) and *IDH1/2*-mutated and *TP53*-mutated AML (resistance). Moreover, *TET2* mutations have been associated with increased response to hypomethylating agents in MDS [28, 29] and AML with low blast count [28]. We believe that our results suggest the persistence during treatment of a minor clone harbouring *DNMT3A*, *IDH2*, and *BCOR* mutations, which was positively selected and progressively expanded, along with the acquisition of a novel *TP53*-mutated subclone. In parallel, *TET2*-mutated blasts were killed by the treatment.

### 3.2. Detection of a Novel 35 Nucleotides Insertion in Exon 22 of the *DNMT3A* Gene in AML

**Figure 2:**
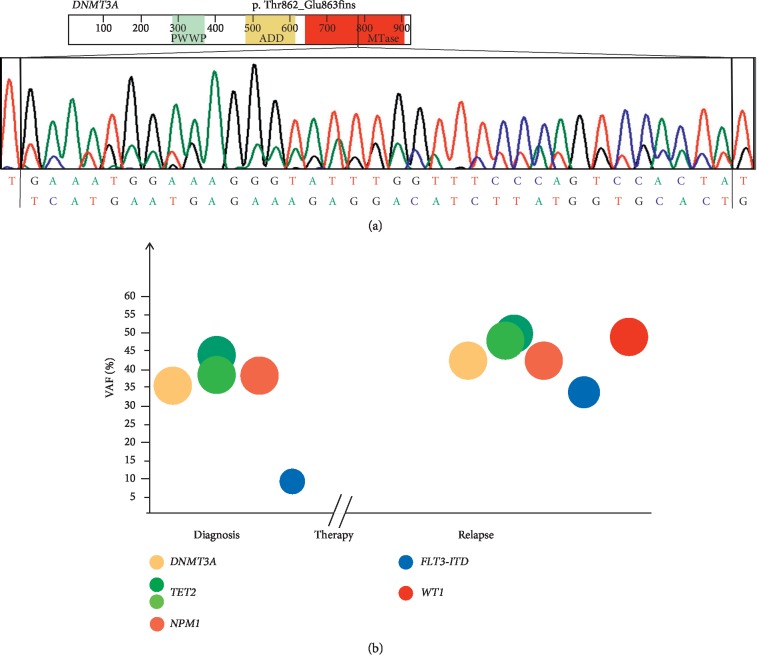
Sanger sequencing of the insertion in the *DNMT3A* gene identified in AML#2 and analysis of clonal evolution based on NGS data. (a) AML#2 showed a novel insertion of 35 nucleotides in the exon 22 of the *DNMT3A* gene (c.2586_2587ins35: TCATGAATGAG AAAGAGGACATCTTATGGTGCAC; p Thr862_Glu863fsins). (b) Representation of clonal evolution of AML#2 from diagnosis to relapse (that occurred after 7 months of complete remission achieved with FLAI-5 chemotherapy regimen).

Targeted deep sequencing leads to the identification of an additional *DNMT3A* genetic alteration consisting in a new insertion of 35 nucleotides in exon 22 of the *DNMT3A* gene causing the amino acid change Glu863Ser followed by a premature STOP codon (c.2586_2587ins35:TC ATGAATGAGAAAGAGGACATCTTATGGTGCAC; p. Thr862_Glu863fsins, Figure 2(a)). The variant, which has never been reported before, was detected in primary leukemic cells isolated from a 63-year-old woman (AML#2), with 80% of blasts in the BM at diagnosis and 90% at relapse. The patient had normal karyotype, low *FLT3*-ITD allelic burden (c.1747_1748ins57: GCTCCTCAGATAATGAGTACTTCTACGTTGATTTCAGAGAATATGAATATGATCCAA; VAF: 11.7% at diagnosis), and intermediate cytogenetic risk according to the ELN 2017 classification [24]. Molecular analysis also detected mutations of *NPM1* (c.863_864insCTTG; p.Trp288fs; VAF 37.4%) and *TET2* (c.395delA; p.Asn132fs; VAF 38.6% and c.5504delG; p.Gly1835fs; VAF 43.6%, Figure 2(b) and Table 2). The patient started fludarabine, arabinosyl cytosine, and idarubicin (FLAI-5) induction regimen [30, 31] and obtained complete hematological remission one month later, with undetectable *FLT3* mutations. After 7 months of complete remission, the patient relapsed. Mutational analysis performed on the diagnosis and relapse samples showed that the *DNMT3A* mutation loads were, respectively, 35.8% and 42.5%. The relapse sample presented an increase of 5.5% for VAF of *NPM1* mutation, along with an expanded *FLT3*-ITD clone (11.7% to 40.1%), the persistence of *TET2* mutations with a slight VAF increase (c.395delA; p.Asn132fs; VAF 48.4% and c.5504delG; p.Gly1835fs; VAF 49.7%), acquisition of a *WT1* mutation (c.1109G > C; p.Arg370Pro; VAF: 48.3%; Figure 2(b) and Table 2). In this patient, the treatment was not able to eradicate the leukemic clone harbouring *TET2*, *DNMT3A,* and *NPM1* mutations, which seems to have acquired an additional *WT1* mutation at relapse. Moreover, AML relapse was characterized by the expansion of the *FLT3*-ITD clone that was reduced but not eradicated by the treatment, with an increase of allelic ratio from 0.09 (diagnosis) to 0.21 (relapse). A recent study showed that the persistence of *DNMT3A* mutation at a VAF ≥2% at first remission is a common event in AML, occurring in 65% of cases with mutation at diagnosis and is associated with older age and inferior relapse-free survival [32].

### 3.3. The Identified *DNMT3A* Mutations Alter DNA CpG Islands Methylation and Protein Stability

**Figure 3:**
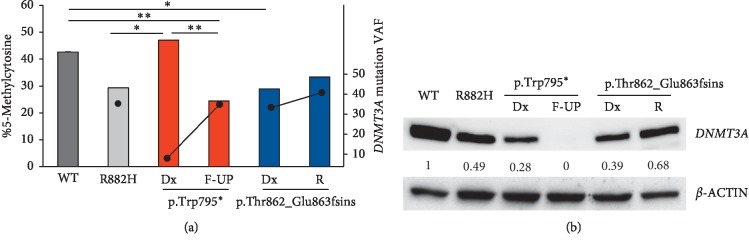
Effect of *DNMT3A* mutations on protein function and expression. (a) CpG islands methylation analysis. The bars represent mean ± standard deviation (SD) of the absolute 5-mC level (percentage of 5-mC left axis; SD values are barely detectable due to low values). Dots represent the *DNMT3A* VAF in the analyzed samples (right axis). A BM sample from an AML patient harbouring the *DNMT3A* R882H mutation (VAF 37.0%) was used for comparison (wt: wildtype; R882H: canonical mutation; Dx: diagnosis; F-UP: follow-up; R: relapse). (b) Western blot analyses of DNMT3A expression in representative AML cases with wt-*DNMT3A* and R882H mutation, followed by diagnosis and follow-up of AML#1, diagnosis and relapse of AML#2 (wt: wildtype; Dx: diagnosis; F-UP: follow-up; R: relapse). *β*-actin was used for loading normalization and quantification. Numbers represent DNMT3A levels normalized on the wt sample.

To investigate the functional consequences of the identified mutations, we performed methylation analysis of CpG islands on DNA extracted from primary leukemic samples of both patients. Our cases showed DNA hypomethylation in comparison with primary AML samples with wt-*DNMT3A* gene or R882H mutation (VAF 37.0%) (Figure 3(a)). In the sample carrying Trp795^*∗*^ mutation, CpG islands methylation shifted from 47.1% at diagnosis to 24.4% at follow-up (*p*=0.020), in accordance with the increase in the mutations allelic burden (from 4.5% at diagnosis to 38.2% at follow-up). At follow-up, CpG methylathion was significantly reduced compared with the wt-*DNMT3A* sample (*p*=0.017). However, the observed hypomethylation status may be associated both with hypomethylating agent treatment and with the expansion of the *DNMT3A*-mutated clone during treatment.

In the sample carrying the insertion at Thr862_Glu863, CpG island methylation at diagnosis was significantly reduced compared with the wt-*DNMT3A* sample (*p*=0.047). Moreover, its levels were similar to those observed in the R882H sample. No major changes occurred between disease diagnosis and relapse in terms of CpG methylation level and *DNMT3A* (c.2586_2587ins35; p. Thr862_Glu863fsins) VAF. The results suggest that the identified mutations induce DNMT3A loss of function, similarly to the R882H alteration. It has been recently shown that hypomethylation is an initiating feature of AML with *DNMT3A*^R882^ [33] and that demethylator phenotypes, which are partially related to *DNMT3A* mutational status, have a prognostic role, independent of age and cytogenetic abnormalities [34]. Future analyses of the aberrant DNA methylation pattern may help define specificities compared with *DNMT3A*^R882^ AML and novel potential silenced or activated enhancers involved in leukemogenesis [35].

To understand whether alterations in DNMT3A protein expression were responsible for the observed changes in CpG methylation, we performed western blot on BM samples at different time points. Protein analysis revealed that despite the low *DNMT3A* mutation burden in the diagnosis sample of the case AML#1 (Trp795^*∗*^), DNMT3A protein levels were reduced to 72% and 21% compared with wt-*DNMT3A* and R882H samples, respectively (Figure 3(b)). Furthermore, the increased VAF of truncated *DNMT3A* was associated with no detectable protein in the follow-up sample (Figure 3(b)). Despite the heterozygous mutational status, the wt protein isoform became undetectable. We speculated that the wt protein was unable to form a heterodimer with the truncated DNMT3A form, which may also be unstable, thus causing a premature degradation. This hypothesis is supported by a recent study, which demonstrates that *DNMT3A* truncation mutations have a dominant negative effect with loss of function and haploinsufficiency in AML [12]. On the contrary, DNMT3A protein was detectable in the analyzed samples from AML#2 (p.Thr862_Glu863fsins). In this patient, we observed that DNMT3A levels were reduced in the diagnosis sample compared with the wt one and were similar to those obtained in the R882H-mutated case (Figure 3(b)). DNMT3A expression slightly increased in the relapse sample, showing a 30% reduction in protein level compared with the wt sample. The data suggest that the mutation, which is predicted to introduce a premature STOP codon, interferes with protein expression but does not alter the stability of the wt protein. Future investigation will be useful to demonstrate if the presence of a portion of the catalytic domain in this truncated protein allows it to bind to wt-DNMT3A. Taken together, the protein and methylation analyses indicate that this mutation results in decreased DNMT3A expression and function.

## 4. Conclusions

*DNMT3A* mutations are becoming highly relevant in hematological malignancies, thanks to NGS technologies which allow us to better characterize myeloid disorders. In this study, we presented two mutations of the *DNMT3A* gene never described in AML and investigated their consequences on protein expression and function. Both mutations localized in the catalytic domain of the DNMT3A protein and were predicted to cause loss of function. The first one is responsible for a truncated, nondetectable protein associated with hypomethylation of CpG islands, which expanded under the pressure of hypomethylating agent treatment. The second mutation was an insertion of 35 nucleotides, with a hypomethylation pattern, suggesting a negative effect on CpG methylation mediated by mutant DNMT3A. The *DNMT3A*-mutated clone escaped therapy selection and likely acquired a *WT1* mutation. Both patients showed evidence of clonal evolution, resulting from the selective pressure induced by treatment. Our strategy, based on bulk sequencing, allows us to draw a picture of AML-related mutations, along with their frequency and to speculate on mutation co-occurrence at disease diagnosis and progression. However, a single-cell sequencing approach is needed to precisely evaluate the clonal complexity of AML across the different disease stages, drive conclusions on the clonal and subclonal architecture, and uncover genomic trajectories of leukemia evolution [36–39]. The presence of cooperating mutations and their contribution to the leukemic phenotype along with the identified *DNMT3A* mutations deserve further investigation. Our data highlight the need to characterize and monitor patient-specific genomic alterations in AML, in order to tailor treatments and allow early detection of expanding subclonal population. Moreover, future studies are needed to define the hypomethylation pattern resulting from the described genomic lesions and its cooperation with differentiation stage-specific histone modification in the regulation of the leukemogenic transcriptional program.

## Figures and Tables

**Table 1:** Mutational profile of AML#1 at diagnosis and follow-up.

Pt	Gene	Locus	NM_ID	Exon	Type	Coding	Amino acid change	VAF (%)	Variant effect
AML#1Dx	*DNMT3A*	chr2:25462022	NM_022552.4	20	SNV	c.2385G > A	p.Trp795Ter	4.45	Nonsense
	*TET2*	chr4:106164773	NM_001127208.2	6	SNV	c.3641G > A	p.Arg1214Gln	3.20	Missense
	*IDH2*	chr15:90631837	NM_002168.3	4	SNV	c.515G > A	p.Arg172Lys	6.06	Missense
	*BCOR*	ChrX:39931683	NM_001123385.1	4	INDEL	c.2915_2916insA	p.Tyr972Ter	4.76	Nonsense
AML#1F-UP	*DNMT3A*	chr2:25462022	NM_022552.4	20	SNV	c.2385G > A	p.Trp795Ter	38.20	Nonsense
	*IDH2*	chr15:90631837	NM_002168.3	4	SNV	c.515G > A	p.Arg172Lys	39.80	Missense
	*TP53*	chr17:7578242	NM_000546.5	6	SNV	c.607G > A	p.Val203Met	5.25	Missense
	*BCOR*	chrX:39931683	NM_001123385.1	4	INDEL	c.2915_2916insA	p.Tyr972Ter	42.08	Nonsense

Pt: patient; Dx: diagnosis; F-UP: follow-up; SNV: single-nucleotide variant; INDEL: insertion/deletion; ins: insertion.

**Table 2:** Mutational profile of AML#2 at diagnosis and relapse.

Pt	Gene	Locus	NM_ID	Exon	Type	Coding	Amino acid change	VAF (%)	Variant effect
AML#2Dx	DNMT3A	chr2:25458586	NM_022552.4	22	INDEL	c.2586_2587ins^*∗*^	p.Glu863Ser	35.79	Nonsense
	TET2	chr4:106155491	NM_001127208.2	3	INDEL	c.395delA	p.Asn132fs	38.55	fs del
	TET2	chr4:106197168	NM_001127208.2	11	INDEL	c.5504delG	p.Gly1835fs	43.60	fs del
	NPM1	chr5:170837545	NM_002520.6	11	INDEL	c.863_864insCTTG	p.Trp288fs	37.41	fs ins
	FLT3	chr13:28608308	NM_004119.2	14	INDEL	c.1747_1748ins^*∗∗*^	p.Gly583_Ser584ins^*∗∗∗*^	11.70	Nonfs ins
AML#2R	DNMT3A	chr2:25458586	NM_022552.4	22	INDEL	c.2586_2587ins^*∗*^	p.Glu863Ser	42.45	Nonsense
	TET2	chr4:106155491	NM_001127208.2	3	INDEL	c.395delA	p.Asn132fs	48.34	fs del
	TET2	chr4:106197168	NM_001127208.2	11	INDEL	c.5504delG	p.Gly1835fs	49.65	fs del
	NPM1	chr5:170837545	NM_002520.6	11	INDEL	c.863_864insCTTG	p.Trp288fs	42.87	fs ins
	WT1	chr11:32417943	NM_024426.4	7	SNV	c.1109G > C	p.Arg370Pro	48.25	Missense
	FLT3	chr13:28608308	NM_004119.2	14	INDEL	c.1747_1748ins^*∗∗*^	p.Gly583_Ser584ins^*∗∗∗*^	40.10	Nonfs ins

Pt: patient; Dx: diagnosis; R: relapse; SNV: single-nucleotide variant; INDEL: insertion/deletion; ins: insertion; fs: frameshift; del: deletion; ^*∗*^insertion of 35 nucleotides: TCATGAATGAGAAAGAGGACATCTTATGGTGCAC; ^*∗∗*^insertion of 57 nucelotides: GCTCCTCAGATAATGAGTACTTCTACGTTGATTTCAGAGAATATGAATATGATCCA; ^*∗∗∗*^SerSerAspAsnGluTyrPheTyrValAspPheArgGluTyrGluTyrAspProSer.

## Data Availability

The clinical and molecular data used to support the findings of this study are included within the article.
